# The Role of Dopamine Replacement on the Behavioural Phenotype of Parkinson’s Disease

**DOI:** 10.3233/BEN-2012-120265

**Published:** 2013

**Authors:** Hajar Alobaidi, Hardev Pall

**Affiliations:** ^1^The Michael Trimble Neuropsychiatry Research GroupDepartment of NeuropsychiatryBSMHFT and University of BirminghamBirminghamUK; ^2^Department of NeurologyUHBFT and University of BirminghamBirminghamUK

**Keywords:** Antipsychotic, dopamine agonist, monotherapy, motor symptoms, Parkinson's disease, psychosis

## Abstract

*Objectives:* The pharmacotherapy of Parkinson’s disease (PD) is often challenging as clinicians have to find a favourable balance between the efficacy on motor symptoms and side effect profiles of different dopaminergic medications. We aimed to assess the available evidence on the role of dopamine agonist monotherapy as an alternative to Levodopa in the treatment of motor symptoms of PD, along with the role of dopamine antagonists in the treatment of PD-related psychosis.

*Methods:* We performed a systematic literature review using the databases MEDLINE, EMBASE, PsycINFO and the Cochrane Library Central register of controlled trials. Two searches were performed, ‘Search 1’ extracting trials on dopamine agonists, and ‘Search 2’ on atypical antipsychotics. Eligible studies were Double-blind Randomised Controlled Trials (RCTs) using the Unified Parkinson’s Disease Rating Scale (UPDRS) and the Brief Psychiatric Rating Scale (BPRS) as outcome measures for Search 1 and 2, respectively.

*Results:* 16 relevant RCTs were extracted from the search results. Overall, dopamine agonists were shown to significantly improve UPDRS scores, with a mean percentage improvement of 14.4% compared to −1.9% in the control arm (P value < 0.05)However, their side effect profile illustrated they were associated with twice the incidence of psychotic symptoms in comparison to the controls. The results on the efficacy of atypical antipsychotics for the treatment of PD-related psychosis were not significant.

*Conclusions:* This evidence-based review confirmed that dopamine agonists can be an effective and safe treatment as monotherapy in PD, however psychotic symptoms remain a significant side effect. Atypical antipsychotics may not be relied upon for the correction of these symptoms due to inconsistent results about their efficacy.

## 1. Introduction

Parkinson's Disease (PD) is a neurodegenerative movement disorder affecting 0.2% of the UK population, rising to 4% in over 80 year olds [1]. James Parkinson originally described some of the key motor symptoms in 1817 in 'An Essay of the Shaking palsy [2]; since then, our understanding of the clinical presentations and neurobiological correlates of PD have progressed considerably, and classical motor symptoms are described as a triad of resting tremor, rigidity and bradykinesia. In addition, non-motor symptoms are becoming increasingly recognised, and are often more disabling than the traditional motor symptoms. These commonly reported clinical features include a wide spectrum of neuropsychiatric, autonomic, sensory and gastro-intestinal symptoms [3].

Although PD is largely an idiopathic condition, with uncertain aetiology, its pathophysiology is better understood. PD is known to be primarily a disorder of the basal ganglia, a group of subcortical nuclei playing fundamental roles in the regulation of movement, cognitive and emotional processes, by means of anatomically segregated parallel circuits which are connect-

ISSN 0953-4180/13/$27.50 © 2013 – IOS Press and the authors. All rights reserved ed to the thalamus and cortex [4,5]. Degeneration of dopaminergic neurons within regions of the basal ganglia, namely the pars compacta of the substantia nigra and the striatum, is thought to be the primary cause of the motor symptoms of PD. Effects of this neuropathological process are seen in the skeletomotor circuit, whereby decreased dopamine leads to increased basal ganglia inhibitory output to the thalamus, which causes thalamocortical activity to decrease, resulting in decreased movement. The disease process behind the non-motor symptoms is less well understood, but hypothesised to be due to similar alterations in neurotransmitter pathways, leading to dysfunction within the non-motor basal ganglia circuitries.

In consideration of this established neuropathological model (degeneration of nigrostriatal dopaminergic neurons), the mainstream pharmacological treatment options for patients with PD are concerned with dopamine replacement at the level of the basal ganglia circuits. Levodopa (L-dopa), a dopamine pre-cursor, has been the gold standard treatment for many years [6]. Although its efficacy in improving motor function has been proven, this must be balanced against the development of motor fluctuations and dyskinesias, along with behavioural side effects (dopamine dysregulation syndrome, impulse control disorders) following long-term use [6,7]. Newer dopaminergic therapies, namely dopamine agonists, have an emerging role in the pharmacological armamentarium for PD, because they are not associated with the severe motor complications of L-dopa, and are thought to potentially have neuroprotective effects [8]. Dopamine agonists have often been used as an adjunct treatment to L-dopa [6], and their exact effectiveness when used in monotherapy remains uncertain.

Moreover, a frequent adverse effect of dopaminergic therapy, particularly of dopamine agonists, is psychosis [9]. The observation that dopamine agonists may lead to an increased incidence of psychotic behaviours in the context of PD, is related to the pathophysiology of psychotic symptoms as non-motor symptoms of PD, which has been associated with dopaminergic hyperactivity within mesolimbic and mesocortical pathways [10]. Thus if dopamine agonists are to be used as monotherapy of choice in clinical practice, the efficacy of current antipsychotic medication also needs to be assessed, in order to establish scientifically sound bases for pharmacological modulation of dopaminergic pathways. In the past, typical antipsychotics (neuroleptics) were used to treat PD-related psychosis, thus aggravating Parkinsonian symptoms through their dopamine-blocking effects [11]. The newer atypical antipsychotic agents seem to have a better tolerability profile, as they are thought to selectively antagonise dopamine receptors located at the level of the mesolimbic pathways, while sparing the nigrostriatal receptors [11].

This review aims to assess the role of newer pharmacological agents in the treatment of both motor and non-motor symptoms of PD, specifically assessing the efficacy of non-ergot dopamine agonists monotherapy in controlling motor symptoms, as well as the role of atypical antipsychotics in treating PD-related psychosis. We will also explore the interlinked effects of dopamine agonists in treating Parkinsonian symptoms and causing psychosis, and vice versa for antipsychotics, as this area is under-researched, yet of great significance in current clinical practice.

## 2. Materials and methods

### 2.1. Data sources

A systematic literature review was conducted in accordance with the Prisma guidelines [12] using the databases MEDLINE, EMBASE, PsycINFO, and the Cochrane Library Central register of controlled trials. Two separate searches were performed: 'Search 1' looked at the role of dopamine agonists as monotherapy in early PD, while 'Search 2' assessed the role of atypical antipsychotics in treating PD-related psychosis. The keywords used in Search 1 were "Parkinson's disease", and "de novo" or "initial therapy" or "monotherapy", and "dopamine agonist" or "ropinirole" or "rotigotine" or "pramipexole" or "apomorphine" or "piribedil". Search 2 was conducted using the keywords "Parkinson's disease", and "psychosis" or "hallucination" or "delusion" or "thought disorder", and "atypical antipsychotic" or "clozapine" or "olanzapine" or "risperidone" or "quetiapine". Both searches were limited to articles written in English, describing pharmacological trials conducted on human subjects from 1990 to April 2011. Keywords were searched as subject headings, as well as within the titles and abstracts. Additional articles were searched for by scanning through reference lists of relevant trials and systematic reviews, as well as manually searching the tables of contents of relevant journals such as "Movement Disorders", "Parkinsonism and Related Disorders" and "Parkinson's Disease".

## 3. Data selection

### 3.1. Study type

Search 1 yielded 957 initial results, whereas Search 2 gave 1381 hits. Titles and abstracts were manually scanned and potentially suitable papers were obtained. Where more than one publication about the same trial was available, the most recent one was included. Inclusion and exclusion criteria were then drawn up according to the results found, and applied to allow the most relevant articles to be sought. For both searches, only double-blind Randomised Controlled Trials (RCTs) were included, so that only the best level of evidence was reviewed.

### 3.2. Participants

All studies involved patients with idiopathic PD; studies on patients with other Parkinsonian disorders were excluded. Search 1 focused on studies on patients with early stage PD, classified as Hoehn & Yahr stages 1 to 3. Participants of reviewed studies were previously untreated with dopaminergic therapy for their PD, or were on such therapy for less than 6 months. Patients taking dopaminergic therapy stopped at least 2 weeks before entry into the trial. Studies with participants taking concomitant non-dopaminergic medications for PD, namely amantadine, selegine and anticholinergics, were included, providing they were on stable doses prior to entry in the trial.

Search 2 focused on trials where participants had idiopathic PD plus psychosis or hallucinations, formally diagnosed according to current criteria. Patients enrolled in the reviewed studies stopped taking any antipsychotic therapy a minimum of 2 weeks before entry into the trials. Concomitant use of anti-parkinsonian medication was permitted, providing the patient was on a stable dose before entry into the trial.

### 3.3. Interventions

For the studies retrieved in Search 1, non-ergot dopamine agonists were used in the treatment group, and compared to a group receiving either placebo or a standard treatment (L-dopa). When open-label L-dopa supplementations for severe disability were permitted for ethical reasons, results provided before the use of L-dopa supplementation were used where available. For Search 2, patients were treated with an atypical antipsychotic in the treatment group, compared to either placebo or another atypical antipsychotic. Any dose or method of administration was permitted in both searches.

### 3.4. Outcome measures

All studies included in Search 1 used the Unified Parkinson's Disease Rating Scale (UDPRS) as the scoring system of symptom severity, with the motor, ADL, combined motor and ADL sub-scores or UDPRS total scores. Only studies where data for mean changes from baseline were available, were included. Whenever available, adverse effects from the use of the treatment compared to the control arm were also assessed as a secondary outcome.

Only studies using the Brief Psychiatric Rating Scale (BPRS) were included in Search 2. Mean changes from baseline were included for the total BPRS score, the BPRS psychosis cluster, or individual items within that cluster. In addition, included trials reported the mean changes in baseline for the UDPRS motor score.

## 4. Data analysis

Relevant studies were analysed using the mean change in scores of the rating scales, as well as calculating the percentage changes. The overall mean from all the studies for each rating scale sub-score was also calculated. Furthermore, the frequency of the most common adverse effects were analysed for the studies retrieved by Search 1.

## 5. Results

After applying the above mentioned inclusion and exclusion criteria, 9/957 trials were selected from Search 1 and 7/1381 from Search 2. The characteristics of these trials from Search 1 and 2 are shown in Tables 1 and 2 respectively.

Available results for the changes in UDPRS scores with the use of dopamine agonists are shown in Table 3 and demonstrate that the mean improvement in all subtypes of UDPRS scores was greater for dopamine agonists than the control group: the majority of trials gave statistically significant results (mean percentage improvement of $-14.4\%$ change in score for dopamine agonists, compared to 1.9% for controls; P value $<$ 0.05). Two studies compared the dopamine agonists to L-dopa. Rascol et al. [14] found that L-dopa was significantly more effective than dopamine agonists at improving the motor UPDRS score. Likewise, the Parkinson Study Group [20] found that the mean improvements in total, motor, and ADL UPDRS scores from

Table 1

Clinical trials of dopamine agonists in Parkinson's disease (Search 1)

| Study, year | Location | Sample size | Mean age (years) | Mean PD duration (months) | Male sex (%) | Mean baseline UDPRS | Randomised treatment | Randomised comparison | Delivery method | Dose range (mg/day)* | Treatment duration (months) | Concomitant PD medications |
|---|---|---|---|---|---|---|---|---|---|---|---|---|
| Shannon et al., 1997 [13] | USA | 335 | 62.7 | 21.6 | – | ADL: 8.3<br>Motor: 18.8 | Pramipexole | Placebo | Oral tablet | 0.375–0.45 | 7.75 | Selegine |
| Rascol et al., 2000 [14] | Europe, Israel, Canada | 268 | 63.0 | 29.5 | 60.8 | ADL: 8.0<br>Motor: 21.6 | Ropinirole | L-dopa, Placebo | Oral tablet | Ropinirole: 0.75–24<br>L-dopa: 50–1200 | 60 | L-dopa supplementation |
| Rascol et al., 2006 [15] | Argentina, France, India, Mexico, Portugal, Spain, South Africa | 401 | 62.4 | 24.0 | 60.8 | Motor: 24.5 | Piribedil | Placebo | Oral tablet | 50–300 | 7 | None |
| Sethi et al., 1998 [16] | USA | 147 | 61.9 | 23.5 | 46.0 | Motor: 17.8 | Ropinirole | Placebo | Oral tablet | 0.75–24 | 12 | Selegine |
| Watts et al., 2007 [17] | USA, Canada | 277 | 63.3 | 16.2 | 64.0 | ADL: 8.5<br>Motor: 21.5 | Rotigotine | Placebo | Transdermal patch | 2–6 | 6 | Anticholinergics, MAO–B inhibitors, NMDA antagonists |
| Giladi et al., 2007 [18] | UK, Germany, Israel | 561 | 61.0 | 15.6 | 57.7 | ADL: 9.0<br>Motor: 23.2 | Rotigotine, Ropinirole | Placebo | Rotigotine: Transdermal patch<br>Ropinirole: Oral tablet<br>Placebo: Both | Rotigotine: 2–8<br>Ropinirole: 0.25–5 | 9.25 | Selegine, Amantadine, Anticholinergics |
| The Parkinson Study Group, 2003 [19] | USA | 242 | 61.4 | 15.6 | 63.7 | ADL: 7.1<br>Motor: 19.3<br>Total: 26.4 | Rotigotine | Placebo | Transdermal patch | 4.5–18 | 2.75 | Selegine, Amantadine, Anticholinergics |
| The Parkinson Study Group, 2004 [20] | USA, Canada | 301 | 61.3 | 19.8 | 48.8 | ADL: 8.8<br>Motor: 22.4<br>Total: 32.3 | Pramipexole | L-dopa, Placebo | Oral tablet | Pramipexole: 0.75–4.5<br>L-dopa: 32.5–750 | 48 | L-dopa supplementation, Eldepryl, Selegiline, Amantadine, Anticholinergics, COMT inhibitors |
| The Parkinson Study Group, 1997 [21] | USA | 264 | 61.7 | 23.0 | 64.3 | Sub-total†: 29.2 | Pramipexole | Placebo | Oral tablet | 1.5–6 | 2.5 | Selegiline, Amantadine, Anticholinergics |

Abbreviations: ADL: Activities of Daily Living; MAO-B inhibitors: Monoamine Oxidase B Inhibitors; NMDA antagonist: N-methyl-D-aspartate antagonist; COMT inhibitor: Catechol-O-methyl Transferase inhibitor; '–' : not available/not applicable. *Placebo doses administered were equal to treatment doses; †UDPRS Sub-total is the sum of motor and ADL scores.

Table 2

Clinical trials of atypical antipsychotics for Parkinson's disease-related psychosis (Search 2)

| Study, year | Location | Sample size | Mean age (years) | Mean PD duration (years) | Male sex (%) | Baseline BPRS* | Baseline UDPRS | Randomised treatment | Randomised comparison | Dose range (mg/day)† | Treatment duration (months) |
|---|---|---|---|---|---|---|---|---|---|---|---|
| Ondo et al., 2005 [22] | USA | 31 | 72.5 | 10.5 | 54.8 | Psychosis: 11.0<br>Total: 47.0 | ADL: 32.5<br>Motor: 32.5 | Quetiapine | Placebo | 100–200 | 3 |
| Ellis et al., 2000 [23] | USA | 10 | 74.2 | 9.3 | – | Psychosis: 10.4<br>Total: 38.3 | Motor: 34.7 | Clozapine | Risperidone | Clozapine: 25–100<br>Risperidone: 1–1.5 | 3 |
| Breier et al., 2002 [24] | USA | 83 | 72.6 | 11.8 | 69.8 | Psychosis: 4.3<br>Total: 18.2 | Motor: 20.6<br>Total: 48.1 | Olanzapine | Placebo | 2.5–15 | 1 |
| | Europe | 77 | 70.7 | 12.6 | 65.8 | Psychosis: 4.0<br>Total: 19.3 | Motor: 20.0<br>Total: 46.7 | Olanzapine | Placebo | 2.5–15 | 1 |
| Shotbolt et al., 2009 [25] | UK | 24 | 72.0 | 8.5 | 66.7 | Total: 40.4 | Motor: 30.1<br>Total: 59.2 | Quetiapine | Placebo | 25–150 | 1.5 |
| Rabey et al., 2006 [26] | Israel | 58 | 75.0 | 10.6 | 56.9 | Total: 35.1 | Motor: 38.3<br>Total: 67.1 | Quetiapine | Placebo | 70–210 | 3 |
| Goetz et al., 2000 [27] | USA | 15 | 72.1 | 13.5 | 46.7 | Total: 29.1 | Motor: 30.2 | Olanzapine | Clozapine | Clozapine: 6.25–50<br>Olanzapine: 2.5–15 | 2 |
| The Parkinson Study Group, 1999 [28] | USA | 60 | 72.0 | 10.6 | 56.7 | Total: 36.8 | Motor: 35.0<br>Total: 56.7 | Clozapine | Placebo | 6.25–50 | 14 |

Abbreviations: ADL: Activities of Daily Living; '–': not available/not applicable; *Psychosis cluster included scores for individual items e.g. Hallucination scores; †Administered placebo doses were equal to treatment doses.

Table 3

Effect of dopamine agonists on mean UPDRS score change

| Study | Drug | Completed study (%) | Mean UPDRS change, no. (%)* | | | | | | | |
|---|---|---|---|---|---|---|---|---|---|---|
| | | | Part II (ADL) | P value | Part III (Motor) | P value | Sub-total | P value | Total | P value |
| Shannon et al., 1997 [13] | Pramipexole | 83 | −1.8 (−22.0) | < 0.0001 | −4.7 (−25.0) | < 0.0001 | – | – | – | – |
| | Placebo | 80 | 0.4 (4.8) | – | 1.3 (6.9) | – | – | – | – | – |
| Rascol et al., 2000 [14] | Ropinirole | 47 | 1.6 (20.0) | 0.08 | −0.8 (−3.7) | 0.008 | – | – | – | – |
| | Levodopa | 51 | 0.0 (0.0) | – | −4.8 (−22.1) | – | – | – | – | – |
| Rascol et al., 2006 [15] | Piribedil | 88 | −1.2 (–) | < 0.001 | −4.9 (−18.9) | < 0.0001 | – | – | – | – |
| | Placebo | 80 | 1.5 (–) | – | 2.6 (11.3) | – | – | – | – | – |
| Sethi et al., 1998 [16] | Ropinirole | 62 | – | – | −5.4 (−30.2) | – | – | – | – | – |
| | Placebo | 68 | – | – | 1.4 (7.9) | – | – | – | – | – |
| Watts et al., 2007 [17] | Rotigotine | 78 | −0.4 (−4.7) | 0.01 | −3.6 (−16.6) | 0.02 | – (−15.1) | 0.01 | – | – |
| | Placebo | 84 | 0.9 (10.6) | – | 0.4 (1.8) | – | – (7.3) | – | – | – |
| Giladi et al., 2007 [18] | Rotigotine | 71 | −2.1 (−22.6) | < 0.0001 | −5.2 (−21.8) | < 0.0001 | −7.2 (–) | < 0.0001 | – | – |
| | Ropinirole | 77 | – | – | – | – | −11.0 (–) | – | – | – |
| | Placebo | 72 | −0.1 (−1.1) | – | −2.1 (−9.3) | – | −2.2 (–) | – | – | – |
| The Parkinson Study Group, 2003 [19] | Rotigotine | 67 | – | – | – | – | −3.7 (−14.0) | < 0.05 | – | – |
| | Placebo | 83 | – | – | – | – | −0.3 (−1.1) | – | – | – |
| The Parkinson Study Group, 2004 [20] | Pramipexole | 55 | 1.7 (18.7) | 0.02 | 1.3 (5.8) | 0.001 | – | – | 3.2 (9.8) | 0.003 |
| | Levodopa | 67 | 0.5 (5.9) | – | −3.4 (−15.1) | – | – | – | −2.0 (−6.3) | – |
| The Parkinson Study Group, 1997 [21] | Pramipexole | 81 | – | – | – | – | – | – | −6.4 (−21.8) | < 0.05 |
| | Placebo | 98 | – | – | – | – | – | – | −0.9 (−3.1) | – |
| Mean of all trials | | DA: 70.9<br>Control: 75.9 | DA: −0.9 (−9.6)<br>Control: 0.4 (1.7) | – | DA: −3.7 (−17.4)<br>Control: 0.3 (1.7) | – | DA: −7.3 (−14.6)<br>Control: −1.3 (−3.1) | – | DA: −4.8 (−15.8)<br>Pl = 0.6 (1.6) | – |

Abbreviations: UPDRS: Unified Parkinson's Disease Rating Scale; ADL: Activities of Daily Living; DA: Dopamine Agonist; '–' : not available/not applicable *UPDRS score changes are shown as sub-scores depending on the availability of data from each trial; Negative UPDRS changes denote an improvement in symptom severity; P values given for each UDPRS sub-score, for treatment compared to control, ($P < 0.05$ are significant results).

Table 4
Incidence of common adverse effects of dopaminergic therapy

| Study | Drugs | Psychotic symptoms, no. (%) | Dyskinesia (%) | Somnolence (%) | Nausea (%) | Dizziness (%) |
|---|---|---|---|---|---|---|
| Shannon et al., 1997 [13] | Pramipexole | – (11.5) | – | 18.3 | 39.0 | – |
| | Placebo | – (2.3) | – | 8.8 | 20.5 | – |
| Rascol et al., 2000 [14] | Ropinirole | 31 (17.3) | 20.3 | 49.0 | 87.0 | 20.0 |
| | Levodopa | 5 (5.6) | 45.5 | 17.0 | 44.0 | 19.0 |
| Rascol et al., 2006 [15] | Piribedil | 46 (23.0) | – | 6.0 | 12.0 | 7.5 |
| | Placebo | 36 (18.0) | – | 2.9 | 3.9 | 4.4 |
| Sethi et al., 1998 [16] | Ropinirole | 5 (7.1) | – | 12.9 | 8.6 | 11.4 |
| | Placebo | 2 (2.6) | – | 03.0 | 2.6 | 6.5 |
| Watts et al., 2007 [17] | Rotigotine | 0 (0.0) | – | 33.0 | 41.0 | 19.0 |
| | Placebo | – (1.0) | – | 20.0 | 17.0 | 13.0 |
| Giladi et al., 2007 [18] | Rotigotine | – | – | 10.7 | 13.5 | 36.8 |
| | Ropinirole | – | – | 12.3 | 15.8 | 3.1 |
| | Placebo | – | – | 16.9 | 13.6 | 8.5 |
| The Parkinson Study Group, 2003 [19] | Rotigotine | 3 (1.5) | – | 22.0 | 47.0 | 24.0 |
| | Placebo | 0 (0.0) | – | 4.0 | 15.0 | 13.0 |
| The Parkinson Study Group, 2004 [20] | Pramipexole | 22 (14.6) | 24.5 | 36.4 | – | – |
| | Levodopa | 12 (8.0) | 54.0 | 21.3 | – | – |
| The Parkinson Study Group, 1997 [21] | Pramipexole | 14 (6.6) | – | 27.2 | 19.7 | 18.3 |
| | Placebo | 0 (0.0) | – | 13.7 | 9.8 | 19.6 |
| Mean values | – | DA: 20.2 (10.2)<br>Control: 9.2 (4.7) | DA: 22.4<br>Control: 49.8 | DA: 22.8<br>Control: 12.0 | DA: 31.5<br>Control: 15.8 | DA: 17.5<br>Control: 12.0 |

Abbreviations: DA: Dopamine Agonist; '–': not available/not applicable.

baseline to 48 months were greater in the L-dopa group than in the dopamine agonist group.

The incidence of the most common adverse effects of dopamine agonists and L-dopa is presented in Table 4. As shown, psychotic symptoms occurred in more than twice as many patients on dopamine agonists compared to controls, including patients on L-dopa. The two trials comparing dopamine agonists to L-dopa also assessed the incidence of dyskinesia, and found that this symptom occurred on average more than twice as often in the L-dopa groups in comparison to dopamine agonist groups [14,20].

Table 5 shows the results of Search 2, with focus on the changes in BPRS scores in patients taking antipsychotics for PD-related psychosis. The means for both the total and the psychosis cluster of BPRS scores demonstrate that although antipsychotic administration leads to an improvement from baseline scores, this improvement is often non-significant from a statistical point of view. The effects of atypical antipsychotics on the motor symptoms of PD were also largely non-significant.

## 6. Discussion

This systematic review of 16 RCTs confirms that the use of non-ergot dopamine agonists is highly effective as dopaminergic monotherapy in early PD. Since previous research has highlighted a common side effect of these drugs is psychosis, we additionally looked at the efficacy of currently used atypical antipsychotic medications in treating these symptoms: in this case, the results were generally mixed and non-significant, and thus the role of atypical antipsychotics in patients with PD-related psychosis still lies in uncertainty.

The role of dopamine agonists in improving the motor symptoms of PD is in line with our expectations from clinical practice, previous research and current guidelines [6]. The advantages of dopamine agonists in comparison to L-dopa in terms of efficacy is difficult to reliably determine from our systematic review, since only two trials focused on this aspect, and both favoured L-dopa. However, clinical experience suggests that dopamine agonists' efficacy is comparable to that of L-dopa, and potentially greater, for tremor symptoms only. In addition, the incidence of dyskinesia was approximately less than half in patients taking dopamine agonists as opposed to L-dopa, and since this is a major concern with L-dopa, dopamine agonists have established their role as a suitable alternative for PD monotherapy.

The incidence of psychosis in patients taking dopamine agonists was approximately twice that of the control group, as expected. This demonstrates that psy-

Table 5

Effect of atypical antipsychotics on mean BPRS score change

| Study | Drug | Completed study (%) | Mean BPRS change, no. (%)* | | | | Mean UPDRS Motor change | P value |
|---|---|---|---|---|---|---|---|---|
| | | | Total | P value | Psychosis cluster† | P value | | |
| Ondo et al., 2005 [22] | Quetiapine | 81 | −1.0 (−2.2) | > 0.05 | −1.0 (−20.4) | > 0.05 | −1.0 (−2.9) | > 0.05 |
| | Placebo | 80 | −7.0 (−14.2) | – | −1.6 (−30.2) | – | −3.0 (−9.4) | – |
| Ellis et al., 2000 [23] | Clozapine | 80 | −3.0 (−8.0) | > 0.05 | −1.5 (−15.3) | 0.32 | −1.8 (−5.2) | > 0.05 |
| | Risperidone | 100 | −6.0 (−15.3) | – | −3.8 (−34.5) | 0.004 | 2.4 (6.9) | > 0.05 |
| Breier et al., 2002: US [24] | Olanzapine | – | −2.7 (−14.9) | 0.7 | −7.0 (–) | 0.4 | 2.9 (12.7) | 0.02 |
| | Placebo | – | −3.1 (−17.0) | – | −9.0 (–) | – | −2.4 (−9.7) | – |
| Breier et al., 2002: Europe [24] | Olanzapine | – | −4.3 (−24.0) | 0.7 | −1.0 (−25.0) | 0.4 | 4.4 (14.4) | 0.04 |
| | Placebo | – | −5.5 (−26.7) | – | −1.4 (−35.0) | – | −2.2 (−14.8) | – |
| Shotbolt et al., 2009 [25] | Quetiapine | 36 | −4.2 (−10.7) | > 0.05 | – | – | −3.0 (−9.6) | > 0.05 |
| | Placebo | 69 | −2.5 (−6.0) | – | – | – | 1.1 (3.8) | – |
| Rabey et al., 2006 [26] | Quetiapine | 50 | −0.2 (−0.6) | > 0.05 | – | – | 2.2 (5.9) | > 0.05 |
| | Placebo | 61 | −4.9 (−11.4) | – | – | – | −1.9 (−4.8) | – |
| Goetz et al., 2000 [27] | Olanzapine | 86 | 0.3 (1.1) | 1.0 | – | – | 12.3 (57.5) | 0.02 |
| | Clozapine | 0 | −7.6 (−24.2) | 0.03 | – | – | −6.0 (18.2) | 0.1 |
| The Parkinson Study Group, 1999 [28] | Clozapine | 90 | −9.0 (−25.1) | 0.003 | – | – | −3.6 (−11.0) | 0.3 |
| | Placebo | 90 | −2.6 (−6.9) | – | – | – | −1.8 (−4.9) | – |
| Mean values | | Antipsychotic: 65.4<br>Placebo: 75.0 | Antipsychotic: −3.8 (−12.4)<br>Placebo: −4.3 (−13.7) | – | Antipsychotic: −2.9 (−23.8)<br>Placebo: −4.0 (−32.6) | – | Antipsychotic: 0.9 (8.7)<br>Placebo: −1.7 (−6.6) | – |

Abbreviations: BPRS: Brief Psychiatric Rating Scale; UPDRS: Unified Parkinson's Disease Rating Scale; '–': not available/not applicable; *BPRS score changes are shown as sub-scores depending on the availability of data from each trial; Negative BPRS changes denote an improvement in symptom severity; †Psychosis cluster included scores for individual items e.g. Hallucination scores.

chosis occurs commonly in PD alone, but the rates significantly increase with the use of dopamine agonists. However the percentage of patients completing the study was only 5% less for dopamine agonists than control; thus psychosis, in addition to the other commonly experienced side effects of nausea, dizziness and somnolence, was often not so severe as to warrant discontinuation of the medication.

These findings regarding the role of atypical antipsychotics in treating psychosis in PD are in line with the current NICE guidelines [6], which also acknowledge the uncertain efficacy of the medications and recommend their use only when other management plans have failed. With regards to the drug with most proven efficacy, our systematic review highlights the fact that the only agent which showed to significantly improve the BPRS score in one trial was clozapine, which is also in line with the current guidelines [6]. In clinical practice, the use of clozapine is usually restricted due to its hazardous adverse effect of agranulocytosis, however the trials included in this study lacked sufficient data regarding this aspect. Moreover, 65.4% of the patients in the antipsychotic arm completed the trial, as opposed to 75% of patients in the control arm, indicating that adverse effects may have been present, but did not cause a significantly greater percentage of drop-outs as a result. The only adverse effect addressed in depth by all the reviewed trials was worsening of Parkinsonian symptoms, and the trials overall demonstrated that atypical antipsychotics do not have a significant effect on aggravating motor function in PD, thus confirming their superiority to their predecessors, the typical antipsychotics or neuroleptics.

The reliability of this systematic literature review may be hindered by a range of potential limitations, which need to be acknowledged. The setting of the trial may be a major limitation, as some were in specialist clinics, others in the community, and many did not explicitly mention the actual setting. In general, more severe cases are likely to be seen in specialist clinics, and may respond less well to treatment; however this limitation is unlikely to affect Search 1 since all trials enrolled patients with early-stage PD. Both searches may be limited by the fact that many trials included concomitant medications, although only trials which stated that patients were on stable doses of these medications were included. Finally, the data available from the reviewed trials were not always complete, despite the high methodological standards of the trials.

Limitations specific to Search 1 include some differences in baseline characteristics. Different doses and delivery methods were used across the 9 reviewed trials; however in most cases, trials that assessed the same type of dopamine agonist used similar quantities and delivery methods. Two of the studies permitted the use of open-label L-dopa supplementation to both treatment and control arms, and provided no data prior to this, which may significantly limit the generalisability of their findings. However, L-dopa was only given in cases of severe disability, and since these trials had the longest durations, most patients participated for a significant time without the supplements. Some limitations also may lie with the use of the UPDRS rating scale, as none of the UPDRS sub-scales were used by all 9 trials. As a consequence, our analysis relied on the mean values provided by the trials which used each particular sub-scale, a small number in some cases. However since the majority of trials analysed the motor and ADL sub-scores, and all the scores gave similar and conclusive results, this limitation should not hinder the reliability greatly. Quality of life measures (either generic or disease-specific) are becoming increasingly popular as major outcome measure for treatment interventions in PD, and this should be reflected in future reviews on these topics. Finally, some of the discrepancies in the results might be explained by the fact that the studies had different funding sources (pharmaceutical industries or academies).

Search 2 produced results with significantly smaller sample sizes than Search 1, already rendering it less reliable in terms of statistical power. The inclusion criteria for Search 2 were somewhat wider and less specific than Search 1, due to the overall poorer quality of trials available, which was already evident from the initial search results. The exact definition of psychosis also presented a degree of variability across studies, and was poorly explained in some cases; thus it may be difficult to draw accurate comparisons across such a wide range of possible psychotic symptoms. However all the trials used BPRS scores, and the fact that percentage improvements were calculated in addition to change in score numbers should provide an element of standardisation to the results. The total BPRS score may hold some intrinsic inaccuracies, since it encompasses other behavioural symptoms (sometimes overlapping with psychosis) such as anxiety and depression; thus, it appears that the individual psychosis cluster score of the BPRS may be more accurately relied upon. Another limitation lies with the fact that two of the studies from Search 2 compared atypical antipsychotics against each other rather against placebo, raising the possibility that comparisons made with baseline may lead to exaggerated results.

These potential limitations should not impinge upon the reliability of the overall findings of this systematic literature review, particularly since results are in line with what is expected from other studies and guidelines. However, we suggest that more double-blind RCTs need to be performed in these areas, as many studies available were open-label, and of insufficient methodological quality to be included in an evidence-based review. Larger sample sizes are needed, particularly regarding studies assessing the role of antipsychotics, to allow more reliable conclusions regarding these drugs to be drawn. Some trials could not be included in this review because of the lack of standardised rating instruments. Therefore, it is suggested that all studies should try and incorporate disease-specific instruments with established psychometric properties in order to allow comparisons across different studies.

## 7. Conclusions

We set out to examine the role of the newer and clinically relevant medications, non-ergot dopamine agonists and atypical antipsychotics, in treating motor symptoms and PD-related psychosis, respectively. After carrying out a systematic literature review using a thorough search on medical databases, 16 double-blind RCTs were found. Thorough analysis of these studies confirmed that dopamine agonists are an effective treatment as monotherapy for patients with early-stage PD. Although their efficacy was not always shown to be comparable to that of L-dopa, they seem to be associated with a lower incidence of dyskinesias. However, psychotic symptoms are a common side effect of dopamine agonists, and the efficacy of atypical antipsychotics in their treatment remains uncertain. Although atypicals represent an improvement from the older neuroleptics, as they do not cause significant changes in motor function, further trials on larger sample sizes are needed in order to reliably assess the therapeutic potential of these medications in PD populations.

## References

[1] Y. Ben-Shlomo, The epidemiology of Parkinson's disease, *Baillieres Clin Neurol* **6** (1997), 55–68.

[2] J. Parkinson, An Essay of the Shaking Palsy 1817, *J Neuropsychiatry Clin Neurosci* **14** (2002), 223–236.

[3] K.R. Chaudhuri and A.H. Schapira, Non-motor symptoms of Parkinson's disease: dopaminergic pathophysiology and treatment, *Lancet Neurol* **8** (2009), 464–474.

[4] R.M. Bonelli and J.L. Cummings, Frontal-subcortical circuitry and behaviour, *Dialogues Clin Neurosci* **9** (2007), 141–151.

[5] S. Tekin and J.L. Cummings, Frontal-subcortical neuronal circuits and clinical neuropsychiatry: An update, *J Psychosom Res* **53** (2002), 647–654.

[6] National Collaborating Centre for Chronic Conditions, Parkinson's disease: national clinical guideline for diagnosis and management in primary and secondary care. London: Royal College of Physicians, 2006.

[7] The Parkinson Study Group, Levodopa and the progression of Parkinson's disease, *N Engl J Med* **351** (2004), 2498–2508.

[8] A.H. Schapira, Future Directions in the Treatment of Parkinson's Disease, *Mov Disord* **22**(Suppl 17) (2007), S385–S391.

[9] L.B. Zahodne and H.H. Fernandez, Pathophysiology and treatment of psychosis in Parkinson's disease: A Review, *Drugs Aging* **25** (2008), 665–682.

[10] D.P. Wint, M.S. Okun and H.H. Fernandez, Psychosis in Parkinson's disease, *J Geriatr Psychiatry Neurol* **17** (2004), 127–136.

[11] M. Doraiswamy, W. Martin, A. Metz and J. Deveaugh-Geiss, Psychosis in Parkinson's disease: diagnosis and treatment, *Prog Neuropsychopharmacol Biol Psychiatry* **19** (1995), 835–846.

[12] A. Liberati, D.G. Altman, J. Tetzlaff, C. Mulrow, P.C. Gotzsche, J.P. Ioannidis, M. Clarke, P.J. Devereaux, J. Kleijnen and D. Moher, The PRISMA statement for reporting systematic reviews and meta-analyses of studies that evaluate health care interventions: explanation and elaboration, *PLoS Med* **6** (2009), e1000100.

[13] K.M. Shannon, J.P. Bennett and J.H. Friedman, Efficacy of pramipexole, a novel dopamine agonist , as monotherapy in mild to moderate Parkinson 's disease. The Pramipexole Study Group, *Neurology* **49** (1997), 724–728.

[14] O. Rascol, D.J. Brooks, A.D. Korczyn, P.P. De Deyn, C.E. Clarke and A.E. Lang, A five year study of the incidence of dyskinesia in patients with early Parkinson's disease who were treated with ropinirole or levodopa, *N Engl J Med* **324** (2000), 1484–1491.

[15] O. Rascol, B. Dubois, A.C. Caldas, S. Senn, S. Del Signore and A. Lees, Early piribedil monotherapy of Parkinson's disease: A planned seven-month report of the REGAIN study, *Mov Disord* **21** (2006), 2110–2115.

[16] K.D. Sethi, C.F. O'Brien, J.P. Hammerstad, C.H. Adler, T.L. Davis, R.L. Taylor, J. Sanchez-Ramos, J.M. Bertoni and R.A. Hauser, Ropinirole for the treatment of early Parkinson disease: a 12-month experience, *Arch Neurol* **55** (1998), 1211–1216.

[17] R.L. Watts, J. Jankovic, C. Waters, A. Rajput, B. Boroojerdi and J. Rao, Randomised, double-blind, controlled trial of transdermal rotigotine in early Parkinson disease, *Neurology* **68** (2007), 272–276.

[18] N. Giladi, B. Boroojerdi, A.D. Korczyn, D.J. Burn, C.E. Clarke and A.H. Schapira, Rotigotine transdermal patch in early Parkinson's disease: A randomized, double-blind, controlled study versus placebo and ropinirole, *Mov Disord* **22** (2007), 2398–2404.

[19] The Parkinson Study Group. A controlled trial of rotigotine monotherapy in early Parkinson's disease, *Arch Neurol* **60** (2003), 1721–1728.

[20] The Parkinson Study Group, Pramipexole vs Levodopa as initial treatment for Parkinson Disease, *Arch Neurol* **61** (2004), 1044–1053.

[21] The Parkinson Study Group, Safety and efficacy of pramipexole in early Parkinson disease. A randomized dose-ranging study, *JAMA* **278** (1997), 125–130.
[22] W.G. Ondo, R. Tintner, K.D. Voung, D. Lai and G. Ringholz, Double-blind, placebo-controlled, unforced titration parallel trial of quetiapine for dopaminergic-induced hallucinations in Parkinson's disease, *Mov Disord* **20** (2005), 958–963.
[23] T. Ellis, M. Cudkowicz, P.M. Sexton and J.H. Growdon, Clozapine and risperidone treatment of psychosis in Parkinson 's disease, *J Neuropsychiatry Clin Neurosci* **12** (2000), 364–369.
[24] A. Breier, V.K. Sutton, P.D. Feldman, D.L. Kadam, I. Ferchland, P. Wright and J.H. Friedman, Olanzapine in the treatment of dopamimetic-induced psychosis in patients with Parkinson's disease, *Biol Psychiatry* **52** (2002), 438–445.
[25] P. Shotbolt, M. Samuel, C. Fox and A.S. David, A randomized controlled trial of quetiapine for psychosis in Parkinson 's disease, *Neuropsychiatry Dis Treat* **5** (2009), 327–332.
[26] J.M. Rabey, T. Prokhorov, A. Miniovitz, E. Dobronevsky and C. Klein, Effect of quetiapine in psychotic Parkinson's disease patients: a double-blind labelled study of 3 months' duration, *Mov Disord* **22** (2007), 313–318.
[27] C.G. Goetz, L.M. Blasucci, S. Leurgans and E.J. Pappert, Olanzapine and clozapine: comparative effects on motor function in hallucinating PD patients, *Neurology* **55** (2000), 789–794.
[28] The Parkinson Study group, Low dose clozapine for the treatment of drug-induced psychosis in Parkinson's disease, *N Engl J Med* **340** (1999), 757–763.
